# The history and evolution of HLA typing external proficiency testing schemes in UK NEQAS for H&I

**DOI:** 10.3389/fgene.2023.1272618

**Published:** 2023-09-18

**Authors:** A. De’Ath, M. T. Rees, D. Pritchard

**Affiliations:** UK National External Quality Assessment Service for Histocompatibility and Immunogenetics, Welsh Blood Service, Cardiff, United Kingdom

**Keywords:** external proficiency testing (EPT), external quality assurance (EQA), HLA, genotyping, phenotyping

## Abstract

The UK National External Quality Assessment Service (NEQAS) provide an external proficiency testing (EPT) service for clinical laboratories. UK NEQAS for Histocompatibility and Immunogenetics (H&I) has been providing EPT schemes for over 45 years and has grown during this time to provide 19 EPT schemes. Accurate human leucocyte antigen (HLA) typing is critical to support safe clinical services, including transplantation, therefore high quality, relevant EPT schemes are required as part of a laboratory’s quality assurance. This article reviews the development of the HLA typing EPT schemes, from the first HLA phenotyping scheme in 1975, via the first HLA genotyping scheme in 1992, through to the introduction in 2017 of HLA third field assessment results from next-generation sequencing technology. In addition, the introduction of EPT schemes to cover HLA associated diseases and pharmacogenetic reactions, including HLA-B27, HLA*B*57:01 and HLA-DQ for coeliac disease are discussed. The accuracy of laboratory EPT results for HLA phenotyping are >96% (2018–2022), HLA genotyping >99% (2020–2022), HLA-B27 testing >99% (2018–2022) and B*57:01 testing >99% (2017–2022). However, for HLA genotyping for coeliac disease 22%–46% of laboratories made errors in 2020–2022. On investigation, the high rate of unsatisfactory performance was attributed to laboratories lacking specific knowledge to interpret HLA genotyping results and accurately report HLA types for coeliac disease. A misleading commercial kit insert was also identified. The assessment of scheme results has uncovered several issues which have been addressed with the intention of educating participants and improving clinical services. The UK NEQAS for H&I EPT schemes have evolved over the past four decades to reflect changes in HLA typing technology, laboratory clinical practice and to cover post-analytical interpretative elements of HLA typing.

##  Introduction

The HLA genes are the most polymorphic genes in the human genome. The remarkable allelic diversity of the Class I and II loci has been revealed by molecular genetic analyses, made possible by the development of recombinant DNA technology, chain-termination Sanger sequencing, Polymerase Chain Reaction (PCR) amplification, and more recently next-generation sequencing (NGS). The current understanding of the genetic organisation and polymorphism of this region is built on the pioneering work of the Immunogeneticists who used serological and cellular typing to begin to define the HLA loci and the allelic variants.

Today, HLA typing is typically performed by specialist H&I laboratories providing support for services, comprising solid organ and haematopoietic stem cell transplantation (HSCT) including volunteer stem cell donor registries, platelet refractoriness, HLA disease and pharmacogenetics associations. Accurate laboratory results are therefore critical to guide patient management, through transplant compatibility assessment, disease diagnosis, and directing treatment.

External Proficiency Testing (EPT) or external quality assessment is a critical component of a quality management system and is required by many regulators, including International Organization for Standardization (ISO) 15189 accreditation (Schineider et al., 2017). EPT monitors laboratory performance using “blind” samples intended to simulate clinical equivalents. Laboratory test results are evaluated by the EPT provider and reports issued to participating laboratories detailing their performance. Continued participation in EPT and corrective action in the event of any performance issues supports improved laboratory performance over time. The EPT process helps to ensure that laboratory testing is comparable, safe, and clinically effective no matter where testing is performed.

UK NEQAS for H&I (https://ukneqashandi.org.uk/) is one of 16 EPT providers in Europe (https://efi-web.org/fileadmin/Efi_web/Resource_collection/Procedures/List_of_EPT_Providers_May2019.pdf) and has provided an EPT service for clinical laboratories for over 45 years. From its informal beginnings in the 1970s, with some 30 UK laboratories participating in two EPT schemes, it has continued to grow and develop into a professional, dedicated, ISO:17043 accredited service, providing 19 schemes to more than 300 participant laboratories in over 50 countries worldwide. Throughout this time, the Service has maintained its core values of ensuring laboratory testing quality through continual improvement and education for the benefit of patients.

**FIGURE 1:**
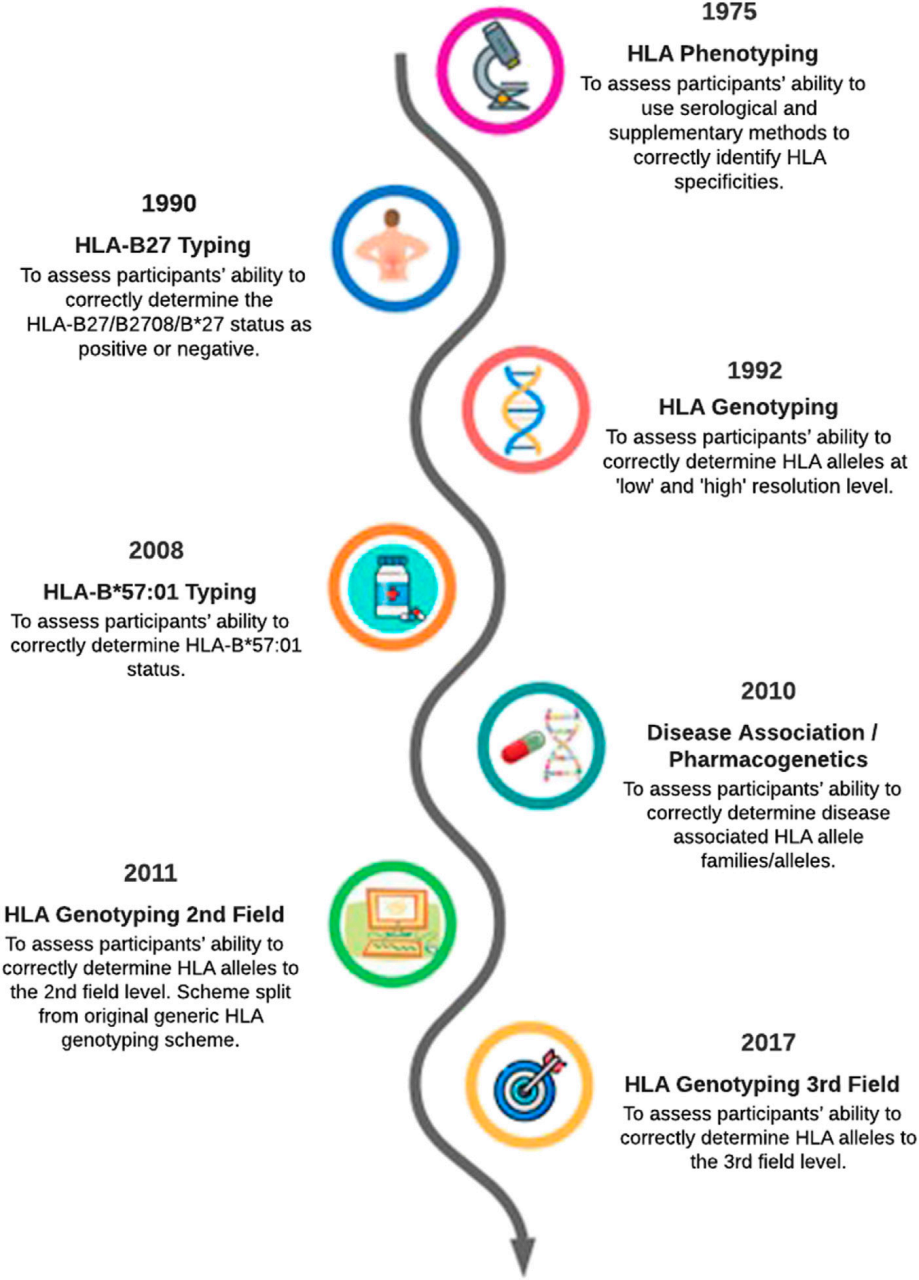
Evolution of UK NEQAS for H&I external proficiency testing schemes relating to HLA typing a simplified timeline.

This article provides an overview of how the EPT service has evolved to reflect changes in HLA typing technology over the past 40 years (Figure 1). The evolution of HLA typing techniques has been reviewed extensively by others (e.g., Dunn, 2011; Erlich, 2012), therefore it not our intention to provide an in-depth review of this aspect. Rather we will highlight some of the technological and clinical milestones in HLA typing to show how the continual evolution of the UK NEQAS for H&I EPT schemes contributes to high quality H&I testing in participant laboratories.

### The first EPT scheme: HLA phenotyping

This first available methodology for HLA typing was serological phenotyping: examining reactions of sera that led to complement activation and cell lysis to determine the HLA type of the lymphocytes being tested. The origin of the UK NEQAS for H&I schemes can be traced back to 1975, when the National Tissue Typing and Reference Laboratory in Bristol initiated a quality control scheme for HLA phenotyping and crossmatching to help laboratories in the United Kingdom and Ireland compare results. This founded the basis of the HLA phenotyping and cytotoxic crossmatching schemes still in use today. The Service joined the UK NEQAS consortium in 1989 and the first international participants joined in 1994. In line with the expansion of participants, UK NEQAS for H&I became a founding member of the European Federation of Immunogenetics EPT Committee in 1998 and has been represented ever since.

In the early days, prior to commercially available kits and reagents, laboratory tests for phenotyping were developed “in-house” using locally sourced anti-sera and complement. Therefore, the EPT exercises also included technical exercises comparing batches of complement and the sensitivity of different methodologies, to promote standardisation of procedures and comparable results between laboratories. In the following four decades, this scheme has undergone minor changes such as the inclusion of new specificities, but the core concept scheme remains. Laboratory practices changed with the introduction of molecular tests and phenotyping was no longer used in isolation to produce an HLA type and therefore supplementary genotyping to confirm serological HLA specificity assignments was introduced in 2009. Today, HLA genotyping has largely superseded phenotyping, which is reflected by a 38% decrease in the number of laboratories participating in the scheme between 2015 and 2022. Nevertheless, recent performance in this scheme is good: in the 5-year period 2018–2022, the accuracy of reported HLA phenotypes was 96.1%, with most errors due to non-analytical issues; 56% due to laboratories reporting a broad instead of a split specificity, and 38% due to the incorrect use of molecular nomenclature. Unfortunately, some sample mix-ups were identified during this period, which highlights the value of continuous EPT testing.

### HLA genotyping schemes

The first UK NEQAS for H&I EPT genotyping scheme was introduced in 1992 after the introduction of DNA based methodology into H&I laboratories (Parham, 1988). Initially the scheme covered Class II (HLA-DRB1 and DQB1) but expanded in 1999 to include Class I. Participants could choose to be assessed at “high” or “low” resolution depending on the level of typing performed. By the mid-1990s most laboratories were using PCR-sequence-specific primer (PCR-SSP) methodology (Olerup and Zetterquist, 1992; Bunce et al., 1995), with the majority using “in-house” developed primers. Indeed, by 1997, 21/27 participants were using PCR-SSP, but only 6 used commercially available primers.

The increasing number of HLA alleles detected during the next decade meant continual development of new primer sets for laboratories using “in-house” methods and increasing complexity in manual interpretation of gel electrophoresis bands. This was reflected in the EPT scheme during the 2000s by a gradual move to commercially available kits and methods [e.g., PCR-sequence-specific oligonucleotide probe (SSOP) and sequence-based typing (SBT)] that included software aided analysis; in 2001 71% of participants were testing using PCR-SSP (41% using “in-house” primers), 26% PCR-SSOP and 3% SBT, compared to 2010 where 29% used PCR-SSP, 41% PCR-SSOP and 30% SBT. Over this time-period there was also an increase in laboratories reporting using multiple techniques (from 16% to 36%), reflecting the increasing complexities of HLA genotyping and limitations of available tests.

With changing HLA genotyping technology came changes to the EPT scheme, e.g., in 2005 HLA-DRB3/4/5, DQA1, DPA1 and DPB1 were added as options for “high resolution” typing assessment. In 2011, the HLA genotyping scheme was split into two separate schemes to provide different samples for “high” and “low” resolution typing (later to become first field and second field resolution schemes). This was in recognition of laboratories moving to different technology for “low” (e.g., to support solid organ transplantation) and “high” (e.g., for HSCT) resolution typing.

In 2016 an “interpretative” scheme for first field HLA genotypes was introduced. This was in recognition of the fact many laboratories performed HLA typing using molecular techniques but converted the results into serological HLA nomenclature for reporting or assessment of donor specific antibodies. This scheme aimed to detect any post-analytical errors due to the conversion between HLA nomenclature systems which could impact on patient care.

With the introduction of NGS and real time-PCR/quantitative-PCR assays into H&I laboratories and changing clinical practice, the HLA genotyping schemes were further modified. In 2017, the option to report HLA genotypes at the third and fourth field was introduced to the second field resolution scheme. The first laboratory reporting EPT samples using NGS was in 2014, using an “in-house” method, but it was not until commercial solutions were widely available that this level of typing became more common-place, and enough laboratories were typing at the third or fourth field level to make EPT assessment possible. The move to NGS for HLA genotyping is evident, with 64% of laboratories using NGS in 2022 and 25% being assessed at the third field.

In 2018, HLA-DPB1 typing was included into the HLA first field genotyping EPT scheme, as many laboratories were now performing HLA-DP genotyping in support of solid organ transplantation. As these laboratories only require a DP type at the resolution to ascertain if a donor-specific antibody is present, the first field genotyping scheme was altered to allow laboratories to report at the resolution that is applicable to their clinical need, including reporting DPB1 alleles that differ at the first field.

Overall, performance in the molecular HLA typing scheme is excellent; in the 3 years 2020–2022 the accuracy of HLA genotyping at the first field resolution was 99.6%, second field 99.7% and third field 99.5%. Errors are often due to post-analytical errors. Continuous improvement in the quality of HLA typing has been noted by other EPT providers (Bogunia-Kubik et al., 2010; Kekik Cinar et al., 2020).

## Educational HLA typing schemes

From the outset the Service has provided educational material to share “interesting” types. In the early days, this was “rare-cell” exchanges and by today’s standards, the samples distributed would not be “interesting”. However, in the 1980s issues assigning A28 in the presence of A2 or detecting Aw33 (A33) highlights some of the challenges that faced the early “tissue typers” and the important role these exchanges provided for laboratories to compare performance of anti-sera with challenging types. The introduction of molecular typing in the 1990s shifted the focus to the detection of rare HLA alleles, or expression variants such null alleles. Over the years, testing of routine EPT samples has contributed to the identification of novel HLA alleles, including A*23:12 (Hammond et al., 2006), A*11:15 (Bendukidze et al., 2006), DQB1*02:01:04 (Smillie et al., 2011), and A*03:162N (Bengtsson et al., 2014).

This educational ethos is still at the core of the Service. Paper-based clinical scenarios in which participants are asked to provide interpretation of results and clinical advice now forms a key component of the educational provision of the Service. Webinars started in 2021 to discuss the results of these scenarios have provided further opportunity for discussion, learning, and sharing of practice between laboratories. In this way the EPT service has moved from solely covering the technical elements of the laboratory testing, to cover appraisal of result interpretation and clinical advice.

## HLA disease association/pharmacogenetic schemes

In 1990, the first HLA-disease association EPT scheme was introduced. This was for HLA-B27 testing, which aids in diagnosis of Ankylosing Spondylitis. The scheme was initially dominated by cytotoxic methodology, but by the mid 1990s flow-cytometry and molecular based methodology had started to replace phenotyping for HLA-B27 testing; in 1996 50% of laboratories used phenotyping, 38% flow cytometry and 14% a molecular technique. Despite the move to other technologies, even as recently as 2016, some laboratories still reported HLA-B27 results using serological cytotoxic methodology, although the proportions are much reduced; 6% phenotyping, 62% molecular and 33% flow cytometry.

The overall performance of laboratories for HLA-B27 typing is excellent with 99.4% of samples correctly assigned (2018–2022) During the 5 years, 48% of samples distributed by UK NEQAS for H&I were HLA-B27 positive, and there was a greater proportion of false negative (67.5%) than false positive results (32.5%). There was no trend with methodology used.

The next targeted HLA typing scheme was for B*57:01 created in 2008 to support testing for Abacavir hypersensitivity. The accurate detection of B*57:01 is crucial; clinicians rely on negative reports to prescribe Abacavir or withhold it for positive patients to avoid potentially life-threatening hypersensitivity reactions (Cargnin et al., 2014). In terms of accuracy of testing, in the first 6 years (2008–2013) there were four errors (0.46%), all false negative B*57:01 reports (Darke and Corbin, 2014). In the last 6 years, 2017–2022, there was a 40% increase in participants and 13 errors (0.34%) with 9 false negative results, at least one of which stemmed from a pre-analytical error. A low error rate has also been reported by other EPT providers (Turriziani et al., 2016).

The number of HLA genes identified as being of diagnostic use to avoid hypersensitivity reactions has since expanded. These additional HLA-associated pharmacogenetic reactions, e.g., B*15:02 and carbamazepine (Tangamornsuksan et al., 2013), will be combined with the B*57:01 scheme to make a complete HLA pharmacogenetic EPT scheme.

### The challenges of HLA typing to aid the diagnosis of coeliac disease

The most recent HLA genotyping scheme was developed in 2010 to aid disease diagnosis. Initially it covered Class II HLA genotyping only, notably for HLA-DQ2/DQ8 for Coeliac Disease (CD) and DQ6 for Narcolepsy but was expanded in 2018 to cover Class I associated diseases. This dedicated HLA disease association scheme is primarily aimed at laboratories that perform partial HLA typing or use commercial kits to detect the presence or absence of specific disease-associated HLA alleles, to support diagnosis of, e.g., CD, Birdshot Retinopathy, Actinic Prurigo, Psoriasis and Narcolepsy. This scheme also allows laboratories to report interpretative comments, but these are not currently assessed.

The scheme is highly flexible; laboratories can register for the diseases relevant to their repertoire and report their results at the resolution that is reported to their clinical users. Results are assessed by comparing participant results to a reference HLA type. The absence of a prescriptive reporting format or resolution allows the scheme to mimic the way a laboratory reports clinically. There have been an uncharacteristically high number of errors in the scheme (De’Ath and Rees, 2019) compared to other UK NEQAS for H&I schemes, with 22%–46% of laboratories making errors in the past 3 years (2020–2022). Performance in relation to HLA typing for CD is especially substandard, likely due to the complexity from multiple genes relating to the specific DQ heterodimers (encoded by DQA1 and DQB1) which confer susceptibility to CD. Suboptimal performance in CD schemes has also been noted by other EPT providers (Horan et al., 2010).

There are several issues likely contributing to the high number of errors in this scheme. The availability of “pos/neg” commercial kits for HLA associated diseases has increased the number of non-specialist laboratories performing this testing. Many of the errors appear to be due to a lack of understating of HLA nomenclature, reflected by the finding that 71% of laboratories with HLA genotyping errors for CD over the past 5 years were non-specialist laboratories. For example, a sample with a reference type of HLA-DQB1*03:01, -; DQA1*05:05,—was reported by one participant as “half DQ2 positive.” When questioned the laboratory indicated that they wished to convey that they had found DQA1*05 in the sample but not DQB1*02. Laboratories not understanding the detection capabilities or resolution of results provided by commercial kits is another common reason for errors, highlighting that a full understanding of a kit is required to interpret and report the correct results.

Many laboratories with personnel not trained in H&I struggle understanding DQB and DQA subunits and their association. For example, a lab noted that their current guidelines are “*to not report DQ2.2 as DQ2 positive but to report it as* “DQ2 negative DQB1*02 positive” *and that risk of coeliac disease cannot be excluded based on genotype.*” This type of reporting is contradictory and not informative for the laboratory’s service users. UK NEQAS for H&I offers support and expertise and works directly with laboratories that report incorrect results to improve their understanding of HLA and CD.

### UK NEQAS for H&I guidelines

The notable lack of standardisation in reporting HLA types in relation to CD (Horan et al., 2010; Tye-Din et al., 2015) and the high proportion of laboratories with errors has prompted UK NEQAS for H&I to develop a set of guidelines on laboratory testing and the clinical interpretation of HLA genotyping results to support a diagnosis of CD (awaiting publication). Assessment of clinical interpretation of results for CD, alongside the reporting of HLA types, using the guidelines as a benchmark for evidence-based responses is planned. The aim is to ultimately harmonise and improve the standard of both testing and reporting of results to clinicians.

### Clinical governance

UK NEQAS for H&I work closely with manufacturers and regulatory agencies to alert them to issues and assist in early resolution of problems with assays, analysers, and test kits. The corrective and preventative action investigations submitted by laboratories with errors in EPT, together with the information on testing methodology can help to highlight potential issues.

**FIGURE 2:**
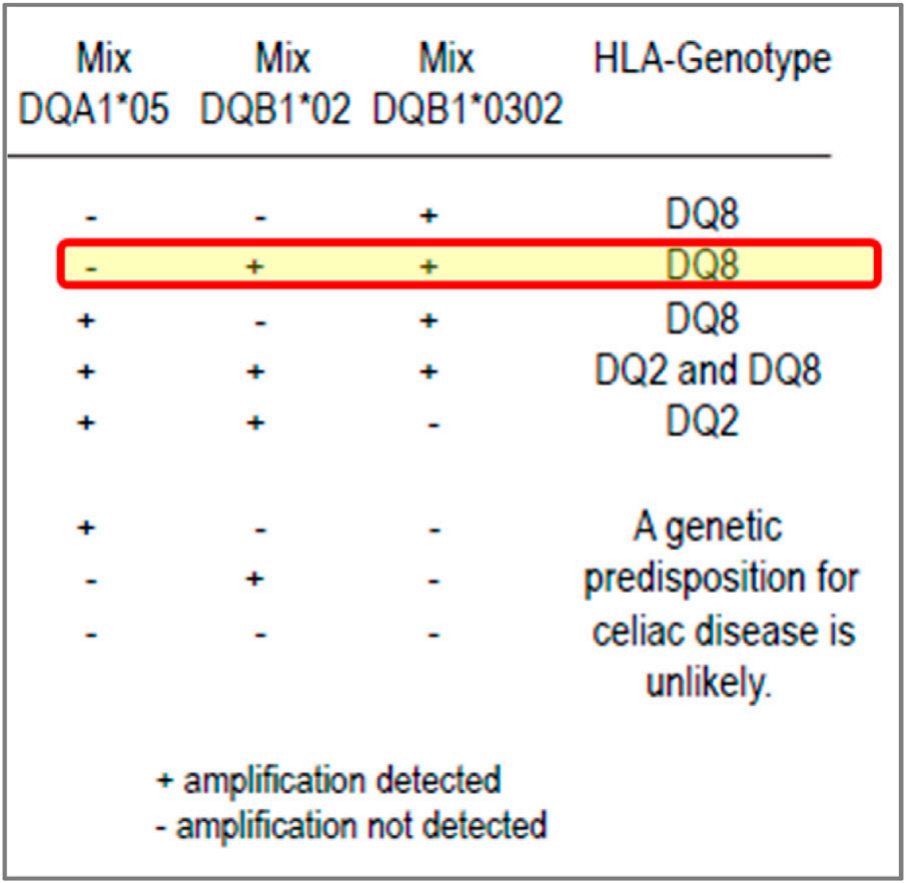
Excerpt from a coeliac disease commercial kit package insert: an example of misleading result interpretation guidance. Insert from a commercial kit for result interpretation of coeliac disease testing. The yellow highlighted row shows the interpretation could be misleading, especially for labs with limited H&I experience. The package insert states it can detect DQA1*05, DQB1*02 (DQ2) and DQB1*03:02 (DQ8) with a positive, negative and internal control. The interpretation of results for the kit suggests that if a user notes a positive reaction in the mixes for DQB1*02 and DQB1*03:02 but negative for DQA1*05, they should assign the DQ8 genotype only even though DQB1*02 (DQ2, but not with DQA1*05) has been defined. Although the relevance of this DQ2 heterodimer is less than the higher risk DQ2.5 heterodimer, it is very misleading for laboratories.

For example, in 2018, two participants reported several HLA genotypes incorrectly for CD. The investigation found an issue with the package insert of the commercial kit being used. These participants, who were not specialist H&I laboratories, relied on the interpretation of results given in the kit package insert and reported the results in accordance with the result interpretation provided the kit, which was incorrect (Figure 2).

UK NEQAS for H&I provided education and support to the laboratories. The Service also contacted the manufacturer to make them aware of the deficiency in their product insert but the company did not respond. The decision was taken, to report the issue to the UK’s Medicines and Healthcare products Regulatory Agency (MHRA). The MHRA contacted the company, and subsequently the manufacturer resolved the issue by redesigning and revising the package insert to add greater clarify on result interpretation. Subsequently, it was noted that laboratories that used this kit were not affected by the same performance issue in subsequent testing. This action resulted in more accurate reporting of the risk of susceptibility to CD for patients.

## Discussion

Laboratory participation in EPT schemes and the subsequent comparison of findings with numerous peer laboratories is an important and unique contribution to a laboratory’s quality assurance programme. EPT providers are uniquely placed with access to large sets of data continually monitored over time. This distinct perspective is invaluable in the support of national and international organisations, and in the production of guidelines and scientific publications. We believe that a notable feature of UK NEQAS is its wider support to participants such as offering training and education through scientific meetings and webinars.

EPT must constantly evolve to provide a responsive service able to adapt to changing clinical practice. EPT providers have a duty to provide schemes that are appropriate to the needs of its service users and ensure the quality of laboratory testing for patients. As testing methods improve so must the scheme designed to assess it, especially in terms of the assessment criteria.

Future EPT considerations for HLA typing will focus on the impact of new technologies such as long read Nanopore sequencing and how this impacts transplantation. Ultimately, laboratory testing strategy will be influenced by clinical requirements so there may be increased interest in HLA typing to the third or even fourth field in the future. Consideration is also required to ensure efficient EPT coverage for new methods which can test for multiple genetic systems. This is particularly relevant in the field of pharmacogenomics where crossover between disciplines will become increasingly evident as laboratories take a “whole genome” approach to testing. The rise of point of care testing (POCT) where testing is performed at/near the site of donor/patient care may also extend to the field of transplantation with future advances in technology, and it will be imperative to ensure the quality of such analytical procedures. This may mean additional considerations for provision of EPT specifically for POCT.

In summary, EPT, like all laboratory testing, is a moving target. By continually evolving and developing schemes UK NEQAS for H&I have aimed to keep pace with the changes in laboratory technology and clinical practice to support laboratory quality assurance. We believe that the Service has achieved this by working with its participants, stakeholders and international organisations with aim to continually develop a service that would be high quality and patient focused.

## Data Availability

The raw data supporting the conclusion of this article will be made available by the authors, without undue reservation.
